# A MIXED METHODS APPROACH TO UNDERSTANDING SPOUSE AND CHILD CAREGIVERS’ EXPERIENCES OF LUCID EPISODES IN DEMENTIA

**DOI:** 10.1093/geroni/igad104.3143

**Published:** 2023-12-21

**Authors:** Kyungmin Kim, Lauren Bangerter, Dawne Finnie, Joseph Gaugler, Lapid Maria, Theresa Frangiosa, Joan Griffin

**Affiliations:** Seoul National University, Seoul, Republic of Korea; Medstar Health, Minneapolis, Minnesota, United States; Mayo Clinic, Rochester, Minnesota, United States; University of Minnesota, Minneapolis, Minnesota, United States; Mayo Clinic, Rochester, Minnesota, United States; Faerge Drinker, Washington, District of Columbia, United States; Mayo Clinic, Rochester, Minnesota, United States

## Abstract

Lucid episodes (LE) in people living with late-stages of dementia (PLWD) have been reported anecdotally, but less is known about how this seemingly unexpected phenomenon is experienced by family members. This study aims to examine variability in family caregivers’ experiences with LE, focusing on the two most common groups of informal caregivers, spouses and children—whether they may exhibit differential appraisals of and responses to LEs. Using a sample of former and current family caregivers from UsAgainstAlzheimer’s A-LIST, we conducted an online survey to spouse and child caregivers (N = 387). Qualitative semi-structured interviews were also conducted with a subset of these caregivers who have witnessed an LE (n = 22). Survey results indicate that child caregivers were more likely to witness a LE. Among “former” caregivers who have witnessed a LE (n = 140), spouses were likely to appraise LEs more negatively and make changes in care decisions (i.e., end-of-life planning and financial decisions) after LE, compared to child caregivers. Among “current” caregivers who have witnessed a LE (n = 80), spouses were more likely to indicate no special circumstances prior to LE, whereas children were more likely to indicate LEs associated with visits from friends and family; there was no difference in positive and negative appraisals of LE between current spouse and child caregivers. Content analysis of qualitative interviews revealed that observed differences between spouses and children were related to their different caregiving contexts, such as relationship history, living arrangement, expectations/motivation, and care resources.

